# Supplementary material for: Learning curve and surgical outcome of robotic assisted colorectal surgery with ERAS program
Source: Sci Rep. 2022 Nov 29;12:20566. doi: 10.1038/s41598-022-24665-w (PMC9709162; doi:10.1038/s41598-022-24665-w)
Supplement: Supplementary file 2 — Supplementary Information 2. [file 41598_2022_24665_MOESM2_ESM.zip › RAL Raw data-Ñ[▒K/062/HS_ñw╝╨░O▒Kñσ.pdf]

正式摘要(電子簽章：0000002113750l4)

W52 / W52 ☐英文標題(注意:無英文姓名-請補病人基本檔!) ☐【讀取】憑證

臺中榮民總醫院

出院病歷摘要

轉入號： 轉出號：

[redacted]

(8)流水編號	
(9)入院日期	110/02/17 (2021) 直腸外科 W52 - 112
(10)轉科日期	
(11)出院日期	110/03/03 (2021) 住院天數計 14 日

診斷

- 入院:R-S colon tumor, adenocarcinoma s/p biopsy
- 出院:R-S colon adenocarcinoma s/p Robot Assisted AR on 2021/2/25

主訴

- Bloody stool for days

病史

- This is a 42 year old patient with history of hepatitis B
  according to the patient, he had symptoms of abdominal cramping pain with bloody stool noted, the patient went to Chen Ching hospital, LGI scope was done and showed R-S colon tumor FAV 50cm and biopsy was done. CT was also done and the patient came to our OPD for second evaluation. This time the patient was admitted for further study.

體檢發現

-
  - General appearance :
    - easy looking , conscious clear , without cardiopulmonary distress
    - Vital signs自動帶入date 體溫(BT) 脈搏(P) 呼吸(R) 血壓(BP) 身高(BH) 體重(BW) BMI
      20210217 36℃ 82次/分 18次/分 119/81mmHg 170cm 71.4kg 24.7
    - Vital signs: B.P. 136/94 mmHg , P.R. 108 /min , R.R. 18 /min , B.T. 37 ℃
  - HEENT :
    - Eyes: no anemic conjunctiva, no icteric sclera, normal EOM
    - ENT: no otorrhea, no rhinorrhea, no erythematous mucosa, no injected tonsil, no deformity
  - Neck: supple, freely movable, trachea midline,no deformity, no engorged jugular vein , no palpable thyroid or lymph node enlargement
  - Chest:
    - Inspection: Grossly normal, symmetric and fully expansion, no accessory muscle used, no operation scar, no spider angiomata, no gynecomastia
    - Palpation: no palpable mass or nodule, no subcutaneous emphysema, no tenderness
    - Ausculation: clear breathing sound
  - Heart:
    - Percussion: normal heart size
    - Ausculation: regular heart beat, normal S1, S2, no gallop, no murmur
  - Abdomen:
    - Inspection: in
      - flat contour, symetrically, no scar, no stria, no dilated veins, no rashes
    - Ausculation: normoactive bowel sound, no abdominal bruits
    - palpation: soft, no muscle guarding or rigidity, no local tenderness, no rebounding pain, no palpable mass, no palpable pulsation
    - Percussion: no shifting dulllness, no hepatomegaly, no splenomagaly

-
Peripheral pulsation :	CA	BA	RA	FA	PA	PTA	DPA
Right side :	++	++	++	++	++	++	++/+++
Left side :	++	++	++	++	++	++	++/+++
    - Genito-anus: no tenderness or palpable mass lesion or rectal shelf, normal external genitalia, normal anal tone, brown stool

CRS CONDITION

-
    - Lung

手術日期及方法

- 手術日期及方法20210225 Robot Assisted Colectomy(L)

住院治療經過

- After admission, colonoscope was repeated. 30cm FAV tumor was noted. PET scan was done and showed no distant metastasis. Pathology of other hospital showed adenocarcinoma, Robotic assited AR was done smoothly on 2021/2/25 . He tried bland TWO diet on 02/27 without abdomen discomfort. We discahrged the patient on 2021/03/03 with stable condition.

合併症

一般檢查

特殊檢查

病理報告

其它

出院時情況

- 15.計劃性出院

出院指示

藥名	劑量	劑量單位	用法	頻次	數量	數量單位	現況	天數	單位
$Biofermin tab	2	TAB	PO	TID	84	TAB	TH	14	天
Ultracet tab	1	TAB	PO	Q6HPRN	28	TAB	TH	7	天

出院計劃

主治醫師 2062F 蔣鋒帆 / 住院醫師 2325G 劉佳樺

正式摘要**(電子簽章：000000211375014)**

W52 / W52

doctorRank:B
